# Bioinformatic analysis and machine learning to identify the diagnostic biomarkers and immune infiltration in adenomyosis

**DOI:** 10.3389/fgene.2022.1082709

**Published:** 2023-01-04

**Authors:** Dan Liu, Xiangjie Yin, Xiaohong Guan, Kunming Li

**Affiliations:** ^1^ Centre for Assisted Reproduction, Shanghai First Maternity and Infant Hospital, School of Medicine, Tongji University, Shanghai, China; ^2^ Department of Obstetrics and Gynecology, Shanghai First Maternity and Infant Hospital, School of Medicine, Tongji University, Shanghai, China

**Keywords:** adenomyosis, bioinformatics analysis, WGCNA, machine learning, diagnostic markers, immune infiltration

## Abstract

**Background:** Adenomyosis is a hormone-dependent benign gynecological disease characterized by the invasion of the endometrium into the myometrium. Women with adenomyosis can suffer from abnormal uterine bleeding, severe pelvic pain, and subfertility or infertility, which can interfere with their quality of life. However, effective diagnostic biomarkers for adenomyosis are currently lacking. The aim of this study is to explore the mechanism of adenomyosis by identifying biomarkers and potential therapeutic targets for adenomyosis and analyzing their correlation with immune infiltration in adenomyosis.

**Methods:** Two datasets, GSE78851 and GSE68870, were downloaded and merged for differential expression analysis and functional enrichment analysis using R software. Weighted gene co-expression network analysis (WGCNA), the least absolute shrinkage and selection operator (LASSO), and support vector machine-recursive feature elimination (SVE-RFE) were combined to explore candidate genes. Quantitative reverse transcriptase PCR (qRT-PCR) was conducted to verify the biomarkers and receiver operating characteristic curve analysis was used to assess the diagnostic value of each biomarker. Single-sample Gene Set Enrichment Analysis (ssGSEA) and CIBERSORT were used to explore immune cell infiltration in adenomyosis and the correlation between diagnostic biomarkers and immune cells.

**Results:** A total of 318 genes were differentially expressed. Through the analysis of differentially expressed genes and WGCNA, we obtained 189 adenomyosis-related genes. After utilizing the LASSO and SVM-RFE algorithms, four hub genes, namely, six-transmembrane epithelial antigen of the prostate-1 (STEAP1), translocase of outer mitochondrial membrane 20 (TOMM20), glycosyltransferase eight domain-containing 2 (GLT8D2), and NME/NM23 family member 5 (NME5) expressed in nucleoside-diphosphate kinase, were identified and verified by qRT-PCR. Immune infiltration analysis indicated that T helper 17 cells, CD56dim natural killer cells, monocytes, and memory B-cell may be associated with the occurrence of adenomyosis. There were significant correlations between the diagnostic biomarkers and immune cells.

**Conclusion:** STEAP1, TOMM20, GLT8D2, and NME5 were identified as potential biomarkers and therapeutic targets for adenomyosis. Immune infiltration may contribute to the onset and progression of adenomyosis.

## Introduction

Adenomyosis is a common hormone-dependent uterine disorder with an incidence of 8%–27% in women of childbearing age (Kissler et al., 2008). It is a benign gynecological disease characterized by invasion of the endometrium into the myometrium. The pathological features of adenomyosis are ectopic endometrial glands and stroma surrounded by the hypertrophic and hyperplastic myometrium, leading to a diffusely enlarged uterus (Bird et al., 1972; Ferenczy, 1998; Benagiano and Brosens, 2006). For many years, the diagnosis of adenomyosis relied on histological examination after hysterectomy. With improvements in imaging techniques, transvaginal ultrasonography and magnetic resonance imaging (MRI) have been proven to be of great value in adenomyosis diagnosis (Stoelinga et al., 2018; Van den Bosch et al., 2019). Adenomyosis can influence the quality of life of women. Women with adenomyosis suffer from abnormal uterine bleeding (AUB), severe pelvic pain, subfertility or infertility, and even asymptomatic symptoms (Gordts et al., 2018). Therefore, it is important to identify the pathogenesis of adenomyosis and explore potential targets for treatment.

Adenomyosis can have a negative impact on pregnancy, from embryo implantation until term. In addition, many patients with reproductive disorders and infertility require assisted reproductive technology (ART) (Vannuccini et al., 2016). Normal endometrial receptivity is important for embryo implantation. However, endometrial dysfunction in adenomyosis may result in low endometrial receptivity and subsequent infertility. The rates of miscarriage and recurrent pregnancy loss are higher in women with adenomyosis (Benaglia et al., 2014; Vercellini et al., 2014; Younes and Tulandi, 2017; Sharma et al., 2019). Adenomyosis is also a potential cause of recurrent implantation failure during *in-vitro* fertilization (IVF) treatment (Tremellen and Russell, 2011; Harmsen et al., 2019). A meta-analysis including nine studies on ART outcomes indicated that the rate of clinical pregnancy was 40.5% *versus* 49.8% and miscarriage rate was 31.9% *versus* 14.1% in women with adenomyosis *versus* without adenomyosis (Vercellini et al., 2014).

The etiology and mechanism of adenomyosis are not fully understood, and several theories have been proposed, including systemic hormonal aberrations, inflammation and metabolic factors. The most two widely accepted theories are 1) tissue injury that occurs at the endometrial–myometrial interface because of endometrial proliferation caused by hypoestrogenism and invagination of the basalis endometrium into the myometrium, implying the importance of the eutopic endometrium, and 2) a *de novo* origin from the metaplasia of embryonic Müllerian remnants or differentiation of endometrial stem/progenitor cells within the myometrium (Budingen and Staudacher, 1987; Chapron et al., 2017; Garcia-Solares et al., 2018; Khan et al., 2022).

With the development of transcriptome analysis, bioinformatic analysis of transcriptome characteristics has been applied to identify the diagnostic markers of diseases (Xiang et al., 2019; Bulun et al., 2021). High expression and hypomethylation of CEBPB are associated with adenomyosis (Xiang et al., 2019). High expression of KCNK9 has been observed in the eutopic and ectopic endometrium of women with adenomyosis (Larricart et al., 1986). Adenomyosis is often considered a chronic inflammatory disease. Many studies have shown that lymphocytes and macrophages increase in the endometrium of women with adenomyosis, accompanied by dysregulated anti-inflammatory and proinflammatory cytokines (Staros, 1988; Ota et al., 1996; Bulmer et al., 1998; Tremellen and Russell, 2012; Bourdon et al., 2021). Immune abnormalities are associated with epithelial–mesenchymal transition, which facilitates the migration of endometrial cells (An et al., 2017). Therefore, it is important to explore the correlation between diagnostic biomarkers of adenomyosis and immune cell infiltration.

In this study, comprehensive bioinformatic analysis and machine learning algorithms were applied to identify the diagnostic biomarkers and explore the immune infiltration in adenomyosis. We downloaded two microarray datasets for adenomyosis from the Gene Expression Omnibus (GEO) database as the metadata cohort. Differential gene expression analysis was performed between the endometrium of women with adenomyosis and those without adenomyosis (control group). Diagnostic biomarkers were identified by integration of the weighted gene co-expression analysis network (WGCNA), least absolute shrinkage and selection operator (LASSO), and support vector machine-recursive feature elimination (SVM-RFE) algorithms. The single-sample Gene Set Enrichment Analysis (ssGSEA) and CIBERSORT were used to identify the different infiltration of immune cells in the endometrium of women with adenomyosis and the control group and the correlation between diagnostic biomarkers and immune cells.

## Materials and methods

### Datasets collection and processing

Human adenomyosis gene expression profiles were obtained from the GEO database (https://www.ncbi.nlm.nih.gov/geo/). Two microarray datasets GSE78851 and GSE68870 were downloaded. The GSE78851 contained expression profile of the endometrium from five women with adenomyosis and three healthy controls, and the platform was Affymetrix Human Gene 1.0 ST Array (GPL6244) (Herndon et al., 2016). The GSE68870 datasets contained expression profiles of the endometrium from four women with adenomyosis and four healthy controls, and the platform was Affymetrix Human Transcriptome Array 2.0 (GPL17586) (Jiang et al., 2016). The two mRNA expression datasets were merged into a single dataset and then normalized. The R package “sva” was used to remove batch effects (Leek et al., 2012).

### Identification of differentially expressed genes and functional enrichment analysis

DEGs in the endometrium of nine women with adenomyosis and seven healthy controls were identified using R package “limma” (Ritchie et al., 2015). The threshold for significant differential expression was set as the false discovery rate-adjusted *p*-value <0.05 and |log2 fold change (FC) | ≥ 1. Functional enrichment analysis was used to explore the functional categories of DEGs. The Gene Ontology (GO) functional analysis was used to explore biological processes (BPs), cellular components (CCs), and molecular functions (MFs) of DEGs, and Kyoto Encyclopedia of Genes and Genomes (KEGG) enrichment analysis (Kanehisa et al., 2008) was applied to explore pathway enrichment analysis. GO and KEGG were performed using R package “clusterProfiler” (Yu et al., 2012), and the significant enrichment was set as *p* < 0.05.

### Weighted gene Co-Expression network analysis

WGCNA is a systematic biological method used to construct gene co-expression networks, cluster genes with similar expression patterns, and explore network modules closely associated with clinical traits (Langfelder and Horvath, 2008). The genes from the GSE68870 and GSE78851 datasets were selected for weighted correlation network analysis using R package “WGCNA”. The co-expression similarity matrix was then transformed into the adjacency matrix by choosing a power of *β* = 7 as the soft-thresholding parameter to ensure an unsigned scale-free network. A topological matrix was then created using the topological overlap measure (TOM) (Langfelder and Horvath, 2008).

To classify genes with similar expression patterns into gene modules, the dynamic hybrid cut method based on TOM-based dissimilarity was performed using the following major parameters: minModuleSize (the minimum number of genes in each module) of 50 and mergeCutHeight (a merging threshold) of 0.2. Therefore, some modules were merged according to the dissimilarity of the estimated module eigengenes, which were defined as the first principal components of a given module and represented gene expression patterns in a module. Finally, the modules with top two positive/negative correlations with clinical traits were chosen as key modules, in which genes with |MM| > 0.8 and |GS| > 0.5 were identified as key module genes. Module membership (MM) represented the correlation of the genes in the module with the module, and gene significance (GS) denoted the correlation of the genes with the trait.

### Screening for candidate diagnostic biomarkers using machine learning algorithms

After intersecting the key module genes identified by WGCNA and DEGs, two machine learning algorithms were used to further screen for significant prognostic genes. LASSO regression is a regression-based algorithm performed through successive shrinking operations that minimize the regression coefficients to reduce the possibility of overfitting (McEligot et al., 2020), thereby reducing redundancy and eliminating irrelevant genes from these analyses (Friedman et al., 2010). LASSO was used to screen for significant prognostic variables with the “glmnet” package in R. The SVM-RFE is a feature selection algorithm used to select the optimal genes to define the minimum classification error and avoid overfitting (Lin et al., 2017; Li et al., 2018). The SVM-RFE was performed to discover the set of genes with the greatest discriminative ability and applied using the “e1071” package. Candidate diagnostic markers were identified by intersecting the genes screened using LASSO and SVM-RFE.

### Collection of clinical samples

Endometrium of women with adenomyosis and those in the control group were collected from hysterectomy specimens at the Department of Obstetrics and Gynecology, Shanghai First Maternity and Infant Hospital. Patients with adenomyosis were diagnosed according to clinical symptoms, such as pelvic pain, AUB, and dysmenorrhea; physical examination results; and imaging reports, including transvaginal ultrasound and MRI reports. The clinical features of these patients with adenomyosis and without adenomyosis have shown in Table 2. The average age of women in the adenomyosis and control groups was 35.83 ± 1.30 *versus* 32.64 ± 1.79, years. Finally, 12 endometrium samples from women with adenomyosis and 11 samples from women without adenomyosis were collected. The endometrium samples were washed with phosphate buffer saline to remove blood and stored an -80 °C for further use. This study was approved by the Ethics Committee of the Shanghai First Maternity and Infant Hospital.

**TABLE 2:** Clinical characteristics of women with adenomyosis and control group for verification.

Variable	AM (n = 12)	CON (n = 11)	P
Age (years)	35.83 ± 1.30	32.64 ± 1.79	0.157^*^
BMI (kg/m^2^)	21.49 ± 0.48	20.95 ± 0.76	0.55^*^

Data are presented as mean ± SEM.

AM: women with adenomyosis; CON: control group; BMI: body mass index.

Student’s *t*-test.

### Verification of candidate diagnostic biomarkers by quantitative reverse transcription polymerase chain reaction

Total RNA was extracted using the TRIzol reagent (Invitrogen, Carlsbad, CA, United States). After the measurement of RNA concentration and quality, approximately 500 ng of total RNA was reverse-transcribed into complementary DNA (cDNA) using the PrimeScript™ RT reagent Kit with gDNA Eraser (Takara, Kyoto, Japan) according to the manufacturer’s instructions. cDNA was used to perform real-time qRT-PCR using the TB Green Premix Ex Taq II (Tli RNaseH Plus; Takara, Kyoto, Japan) on the Applied Biosystems (ABI)7500 Fast Real-time PCR system (Thermos Fisher, MA, United States). The expression levels of actin beta (ACTB), transmembrane protein 97 (TMEM97), glycosyltransferase eight domain-containing 2 (GLT8D2), NME/NM23 family member 5 (NME5) expressed in nucleoside-diphosphate kinase, six-transmembrane epithelial antigen of the prostate-1 (STEAP1), translocase of outer mitochondrial membrane 20 (TOMM20) were detected. The primer sequences are listed in Table 1. Relative quantification of gene expression was performed using the 2^-△△CT^ method. To evaluate the diagnostic efficacy of the biomarkers, the receiver operating characteristic (ROC) curve analysis was performed and the area under the curve (AUC) was calculated using the R package “pROC”.

**TABLE 1:** Primers for qRT-PCR in this study.

Gene name	Sequence (5′-3′)
STEAP1	F: CCC​TTC​TAC​TGG​GCA​CAA​TAC​A
	R: GCA​TGG​CAG​GAA​TAG​TAT​GCT​TT
TOMM20	F: GGT​ACT​GCA​TCT​ACT​TCG​ACC​G
	R: TGG​TCT​ACG​CCC​TTC​TCA​TAT​TC
GLT8D2	F: TGA​CGC​AGA​TGA​TGA​ATC​CGA
	R: TGC​TGT​AGA​TGC​TAT​TGA​TGG​C
NME5	F: CGG​ATT​CAC​CAT​TGT​TCA​GAG​A
	R: CAT​GTA​AGC​TGT​TAA​GTT​GGG​GA
TMEM97	F: TAC​CCA​GTC​GAG​TTT​AGA​AAC​CT
	R: TGT​CAT​GGT​GTG​AAC​AGA​GTA​GA
ACTB	F: TGG​CAC​CCA​GCA​CAA​TGA​A
	R: CTA​AGT​CAT​AGT​CCG​CCT​AGA​AGC​A

### Analysis of immune cell infiltration

The ssGSEA algorithm was used to quantify the immune cell infiltration of 28 immune cells of the adenomyosis gene expression profiles (Bindea et al., 2013; Charoentong et al., 2017). The differential expression levels of 28 immune infiltrating cells in the endometrium of women with adenomyosis and control group were visualized using heatmap and violin plots drawn using the “ggplot2” R package.

### Analysis of correlation between diagnostic biomarkers and infiltrating immune cells

The proportion of 22 immune cells in the different endometrium samples was assessed using the CIBERSORT algorithm. Pearson correlation analysis was used to determine the correlation between diagnostic biomarkers and infiltrating immune cells.

### Statistical analysis

Statistical analyses in this study were performed using SPSS software (version 25.0; IBM, NY, United States) and GraphPad Prism 8.0 (La Jolla, United States). The homogeneity of variance of data was tested using F-test and Brown-Forsythe test. Except otherwise indicated, statistical differences were determined using the Student’s t-test (for normally distributed data) or Mann–Whitney test (for non-normally distributed data). *p* < 0.05 was considered as statistically significant.

## Results

### Identification of DEGs in adenomyosis and functional enrichment analysis of DEGs

**FIGURE 1:**
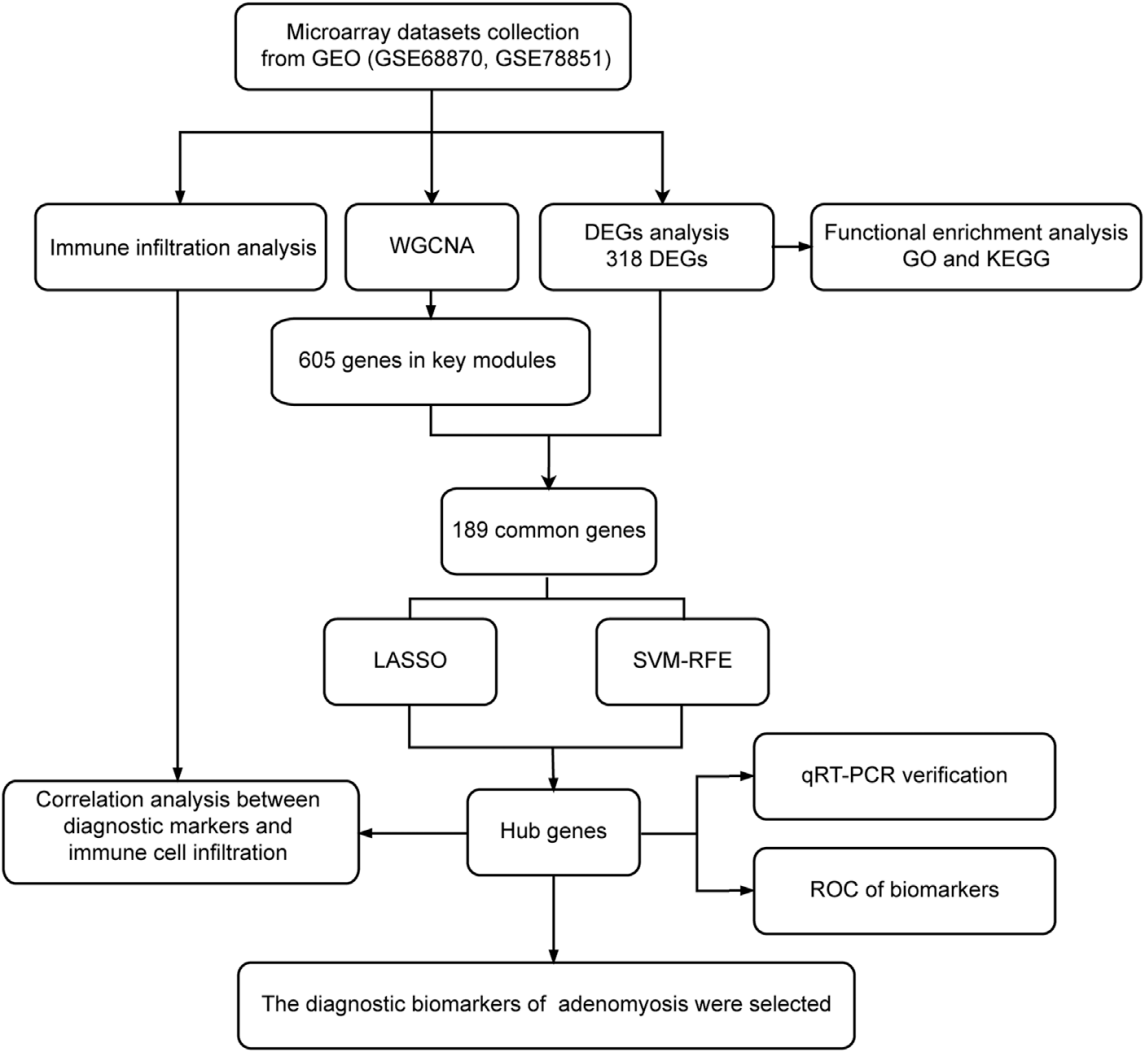
Flowchart of this study. Microarray datasets analysis was conducted for endometrium samples from nine women with adenomyosis and seven healthy controls. DEGs: differentially expressed genes; GEO: Gene Expression Omnibus; GO: Gene Ontology; KEGG: Kyoto Encyclopedia of Genes and Genomes; LASSO: the least absolute shrinkage and selection operator; qRT-PCR: quantitative reverse transcriptase PCR; ROC: receiver operating characteristic; SVM-RFE: the support vector machine-recursive feature elimination; WGCNA: weighted gene co-expression network analysis.

**FIGURE 2:**
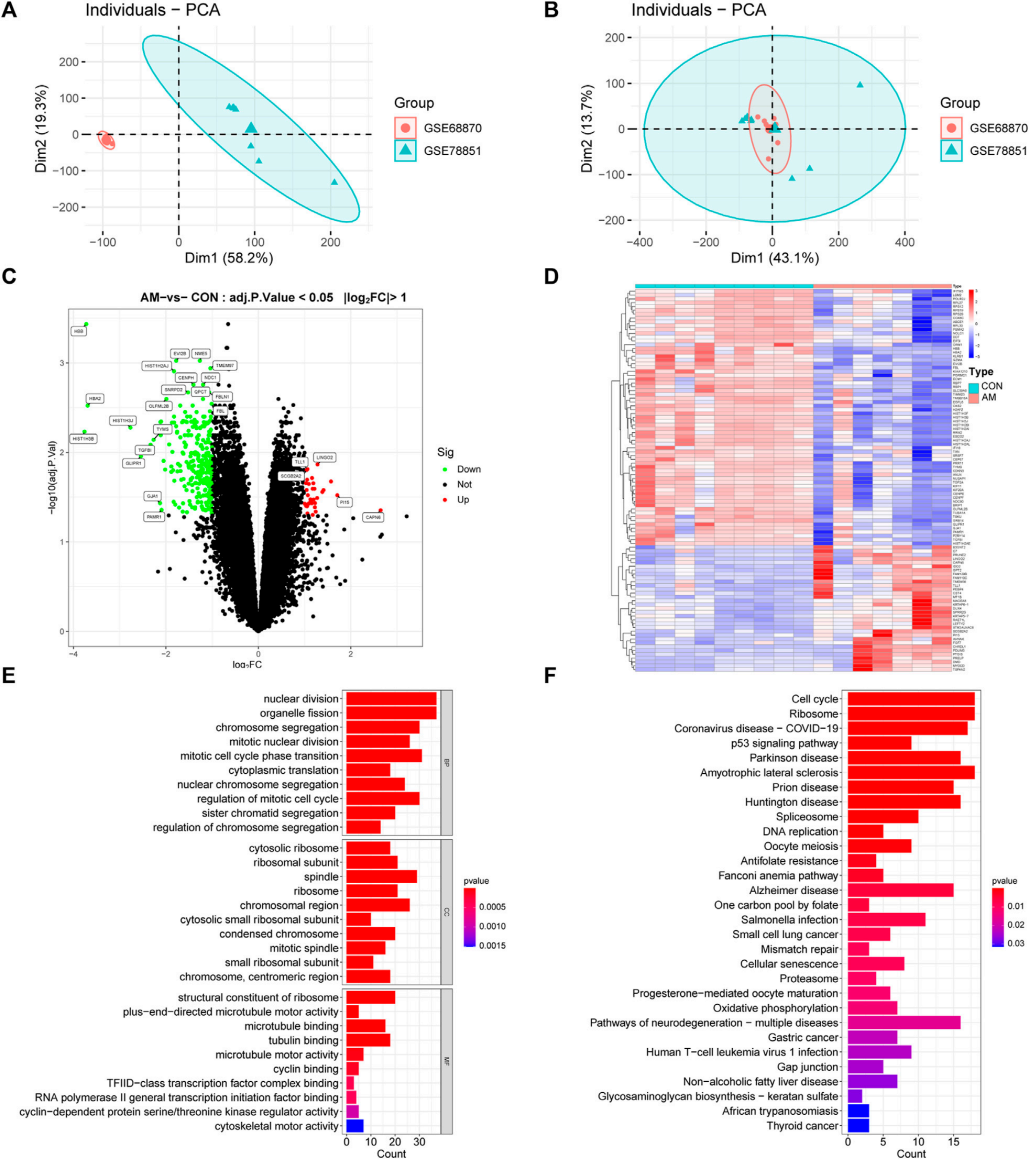
Analysis of DEGs profile in endometrium between women with adenomyosis and controls. **(A)** The PCA plot of sample distribution from the two datasets before removing the batch effects. **(B)** The PCA plot of sample distribution from the two datasets after removing the batch effects. Different colors represent different datasets. **(C)** Volcano plot of DEGs. The red dots represent the up-regulated genes and the green dots represent the down-regulated genes in the adenomyosis group. (|log2 FC | ≥ 1; adjusted *p*-value <0.05). **(D)** Heatmap of DEGs; red indicates upregulated genes and blue indicates downregulated genes in the adenomyosis group. **(E)** GO enrichment analysis of DEGs. The top 10 BP, MF, and CC terms of DEGs. **(F)** The top 30 KEGG pathway enrichment analysis of DEGs. AM: adenomyosis group; BP: biological process; CC: cellular component; CON: control group; DEGs: differentially expressed genes; FC: fold change; GO: Gene Ontology; KEGG: Kyoto Encyclopedia of Genes and Genomes; MF: molecular function; PCA: principal component analysis.

The flowchart of this study is illustrated in Figure 1. After normalization of the merged datasets (GSE78851 and GSE68870), the expression matrix containing 17,203 genes was identified. Figure 2A shows the PCA plot of sample distribution from the two datasets before removing the batch effects using “sva” R package. The red plots represent data from the GSE68870 dataset, and blue plots represent data from the GSE78851 dataset. Samples from the different datasets were distributed separately without intersection. The principal component analysis (PCA) plot after removing batch effects is shown in Figure 2B; the results indicate that the intersection of the two datasets can be used as a batch of data for further analysis. Using “limma” R package, a total of 318 genes, including 33 upregulated genes and 285 downregulated genes, were differentially expressed (adjusted *p* < 0.05 and |log_2_ FC | ≥ 1) between women with adenomyosis and controls. The volcano map shows the upregulated (red dots) and downregulated (green dots) genes in adenomyosis (Figure 2C). The heatmap is shown in Figure 2D.

To investigate the functional and pathway enrichment involved in adenomyosis-related DEGs, we performed the GO and KEGG enrichment analysis. The top 10 BPs, MFs and CCs of GO terms were presented and showed that DEGs were enriched in “nuclear division,” “organelle fission,” “chromosome segregation,” “mitotic nuclear division,” “mitotic cell cycle phase transition,” “cytoplasmic translation,” “nuclear chromosome segregation,” “regulation of mitotic cell cycle,” “sister chromatid segregation,” and “regulation of chromosome segregation” (Figure 2E). The KEGG enrichment analysis showed that DEGs were enriched in “cell cycle,” “ribosome,” “p53 signaling pathway,” “spliceosome,” and “cellular senescence” (Figure 2F). These results indicate the dysfunction of cell cycle, mitosis, and cellular senescence in adenomyosis.

### Weighted gene Co-expression network construction and identification of key modules using WGCNA

**FIGURE 3:**
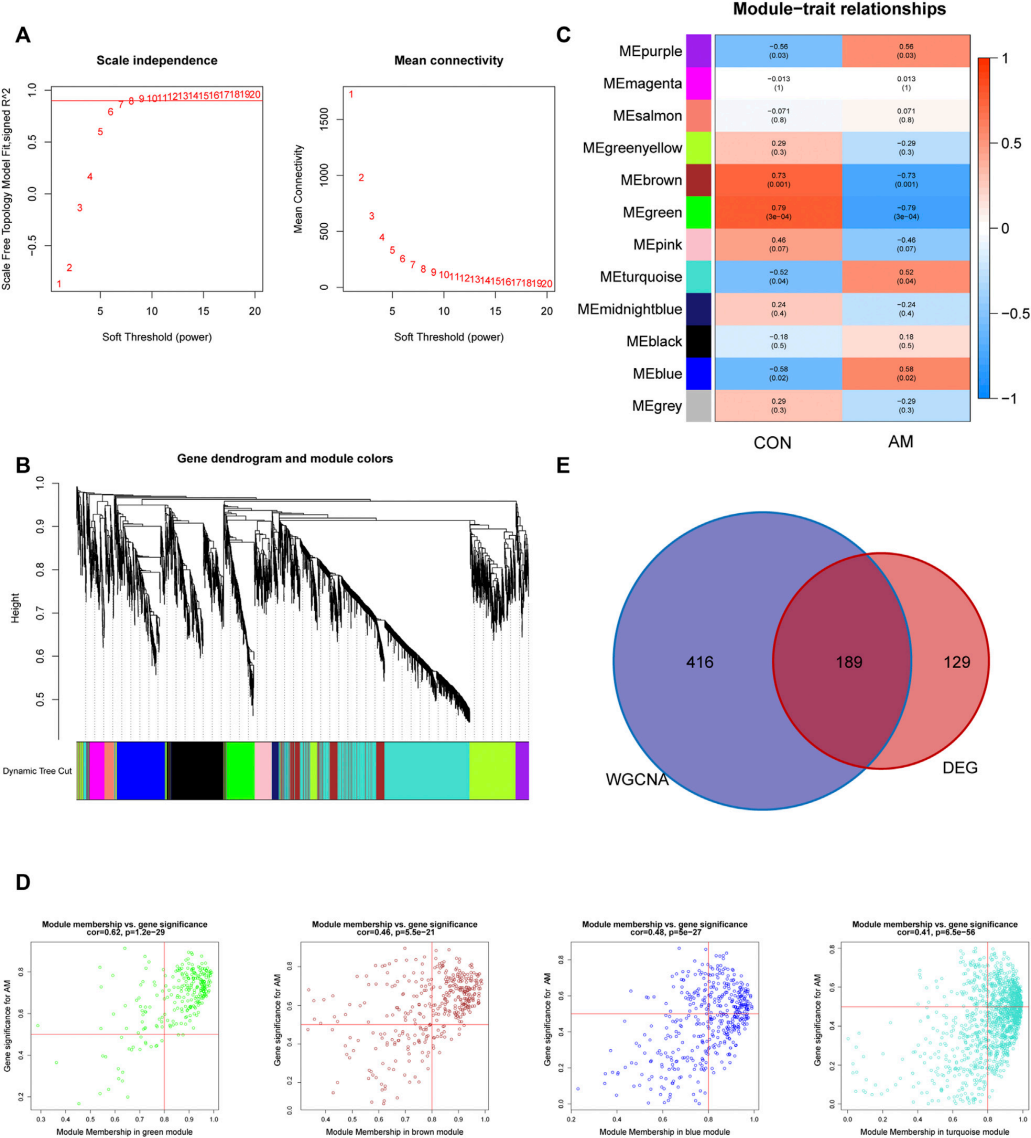
Construction of gene co-expression networks associated with adenomyosis through WGCNA. **(A)** Determination of the soft-thresholding power (*β*). The analysis of the scale-free fit index for different soft-thresholding powers is shown in the left panel, and the mean connectivity for different soft-thresholding powers is shown in the right panel. **(B)** The cluster dendrogram of genes based on the dissimilarity of TOM. **(C)** The heatmap of correlation between genes in different modules and the clinical traits. **(D)** The correlation between GS for AM and MM in four modules (blue, purple, green and brown). One plot represents one gene. The criteria were set as |MM| > 0.8 and |GS| > 0.5. **(E)** Venn diagram of the hub genes obtained by intersecting DEGs and the adenomyosis-related key module genes identified by WGCNA. DEGs: differentially expressed genes; GS: gene significance; MM: module membership; TOM: topological overlap measure; WGCNA: weighted gene co-expression network analysis.

To explore the co-expression networks associated with adenomyosis, WGCNA based on the merged dataset (GSE68870 and GSE78851) was performed to construct the co-expression network. The samples were clustered, and the soft-thresholding power was set to seven when the scale-free *R* ^2^ = 0.9 to ensure a scale-free distribution (Figure 3A). We identified 12 different modules of genes after merging the strongly associated modules using a 0.25 clustering height limit. The cluster dendrogram is shown in Figure 3B. The module–trait relationship showed a correlation between the genes of different modules and clinical traits (Figure 3C). We found that the blue and purple modules had the top two positive correlations with adenomyosis (r = 0.58, *p* = 0.02; r = 0.56, *p* = 0.03, respectively), whereas the green and brown modules had the top two negative correlations with adenomyosis (r = −0.79, *p* = 3 × 10^^−4^; r = −0.73, *p* = 0.001, respectively). Therefore, we chose four modules (blue, purple, green, and brown) showing high correlation with adenomyosis for further analysis. We analyzed the correlation between MM and GS for adenomyosis. After setting the criteria of |MM| > 0.8 and |GS| > 0.5, we identified 605 genes as adenomyosis-related key module genes (Figure 3D). A total of 189 hub genes were obtained by intersecting DEGs and the key module genes (Figure 3E). The 189 hub genes were DEGs showing a high correlation with adenomyosis.

### Identification and verification of diagnostic biomarkers

**FIGURE 4:**
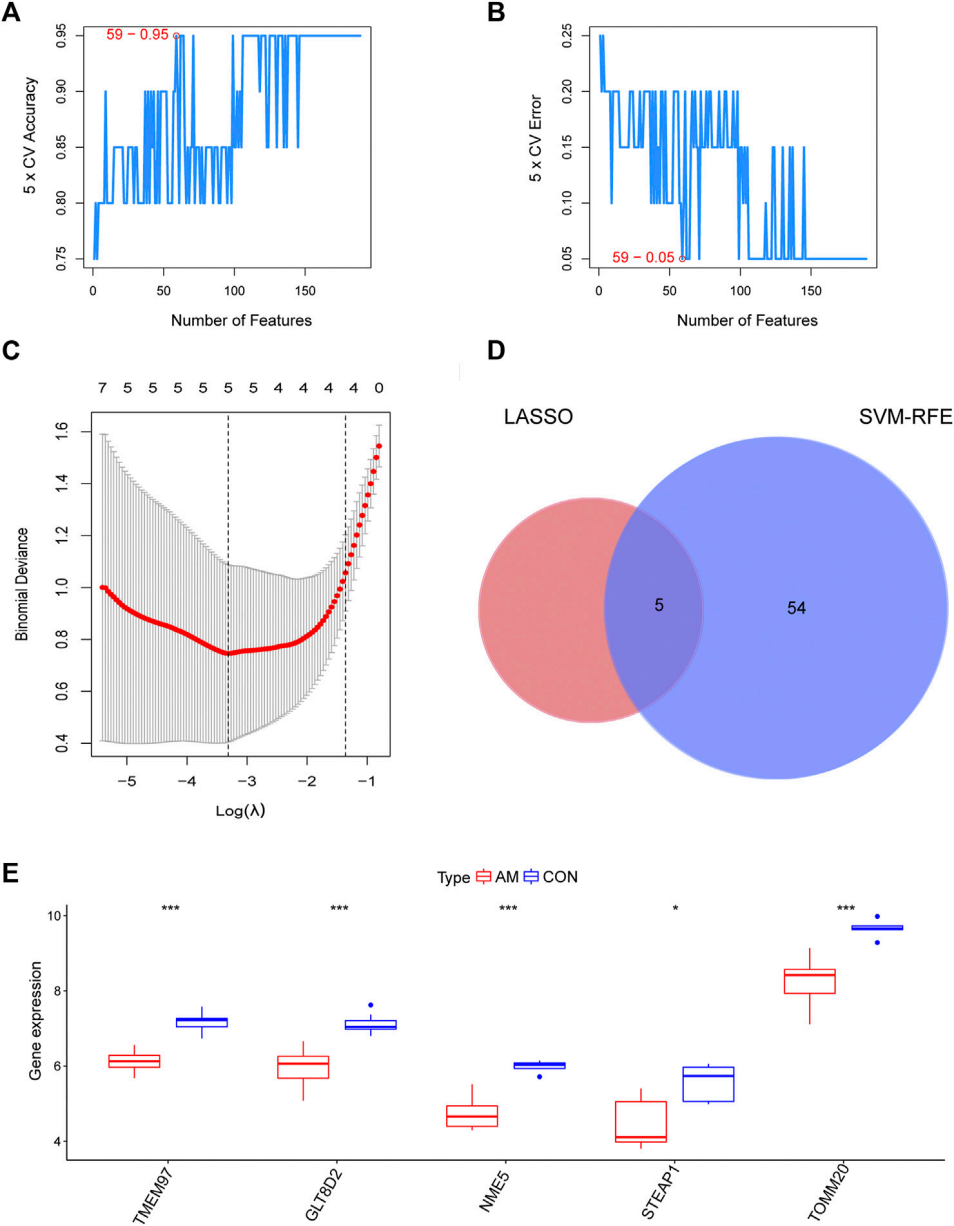
Identification of candidate diagnostic biomarkers by a comprehensive strategy. **(A,B)** Optimal genes identified using the SVM-RFE algorithm. **(C)** Significant prognostic variables screened using the LASSO regression. **(D)** Venn diagram of candidate diagnostic biomarkers screened using LASSO and SVM-RFE. **(E)** The expression of TMEM97, GLT8D2, NME5, STEAP1, and TOMM20 in microarray datasets (*p < 0.05, **p < 0.01, ***p < 0.001, ****p < 0.0001). GLT8D2: glycosyltransferase eight domain-containing two; LASSO: the least absolute shrinkage and selection operator; NDPK: nucleoside-diphosphate kinase; NME5: NME/NM23 family member five expressed in nucleoside-diphosphate kinase; STEAP1: six-transmembrane epithelial antigen of the prostate-1; SVM-RFE: support vector machine-recursive feature elimination; TOMM20: translocase of outer mitochondrial membrane 20; TMEM97: transmembrane protein 97.

**FIGURE 5:**
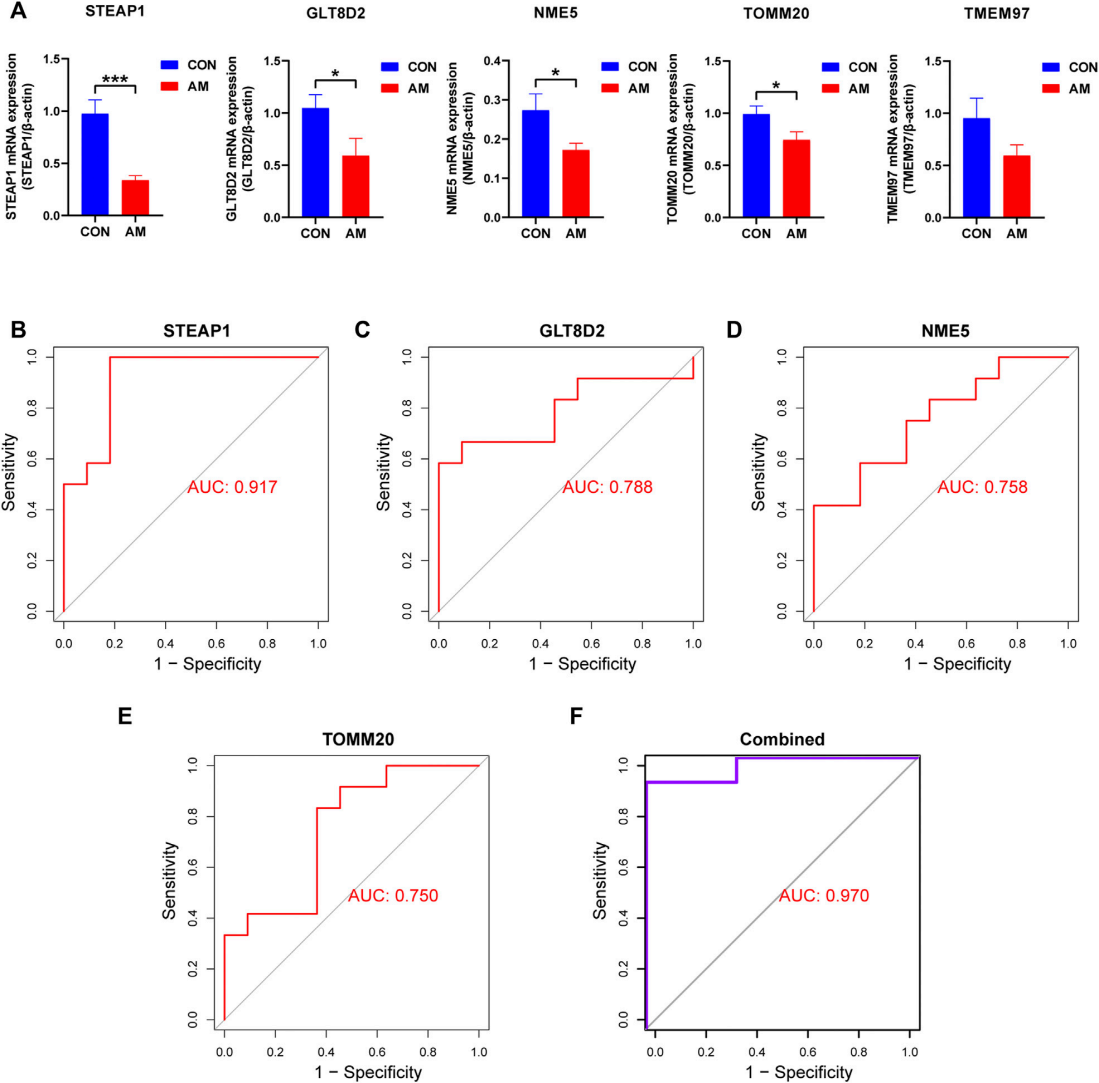
Validation of hub genes using qRT-PCR. **(A)** Validation of the expression of candidate diagnostic biomarkers using qRT-PCR. Four diagnostic biomarkers, namely, STEAP1, GLT8D2, NME5, and TOMM20, were downregulated significantly in the endometrium of women with adenomyosis compared with the control group. The downregulation of TMEM97 did not show statistical significance. **(B–E)** The ROC curve analysis and calculation of the AUC of STEAP1, GLT8D2, NME5, and TOMM20 in the clinical samples. **(F)** The ROC curve to verify the diagnostic efficacy of the combined four diagnostic markers using logistic regression analysis (*p < 0.05, **p < 0.01, ***p < 0.001, ****p < 0.0001). AUC: area under the curve; GLT8D2: glycosyltransferase eight domain-containing two; NDPK: nucleoside-diphosphate kinase; NME5: NME/NM23 family member five expressed in nucleoside-diphosphate kinase; STEAP1: six-transmembrane epithelial antigen of the prostate-1; SVM-RFE: support vector machine-recursive feature elimination; TOMM20: translocase of outer mitochondrial membrane 20; TMEM97: transmembrane protein 97; qRT-PCR: quantitative reverse transcriptase PCR; ROC: receiver operating characteristic.

To screen for candidate diagnostic biomarkers from the 189 hub genes, two different algorithms were applied. The SVM-RFE algorithm was used to identify a subset of 59 features (Figures 4A,B), and the LASSO logistic regression algorithm was used to identify five adenomyosis-related feature variables from 189 hub genes (Figure 4C). Finally, five diagnostic biomarkers, namely, TMEM97, GLT8D2, NME5, STEAP1, and TOMM20, were identified by overlapping the genes screened using the two algorithms (Figure 4D). The expression levels of the five candidate diagnostic biomarkers in the merged dataset are shown in Figure 4E. The microarray dataset analysis revealed that the expression levels of TMEM97, GLT8D2, NME5, STEAP1, and TOMM20 were significantly downregulated in the endometrium of women with adenomyosis compared with those in the control group (*p* < 0.05). These results indicate that the five genes could serve as the diagnostic biomarkers of adenomyosis and the potential targets for therapy. To verify these results, qRT-PCR was used to measure the mRNA expression levels of the diagnostic markers. We collected endometrium samples from 12 women with adenomyosis and 11 without adenomyosis at the Department of Obstetrics and Gynecology, Shanghai First Maternity and Infant Hospital. No significant difference was noted in age or BMI between the groups (Table 2). The expression levels of STEAP1, GLT8D2, NME5, and TOMM20 were downregulated and showed statistical significance (*p* < 0.05) in the adenomyosis group compared with those in the control group. The expression of TMEM97 was downregulated in the adenomyosis group, but without statistical significance (Figure 5A). Therefore, STEAP1, GLT8D2, NME5, and TOMM20 were selected as diagnostic biomarkers. To further validate the diagnostic value of STEAP1, GLT8D2, NME5, and TOMM20, we performed ROC analysis, which revealed that they were valuable diagnostic biomarkers, with AUCs of 0.917, 0.788, 0.758, and 0.750, respectively (Figures 5B–E). In addition, we applied logistic regression analysis to evaluate the diagnostic efficacy of the four biomarkers combined, which revealed that the four biomarkers showed higher diagnostic efficiency when used in combination (AUC = 0.970; Figure 5F).

### Analysis of immune cell infiltration by ssGSEA

**FIGURE 6:**
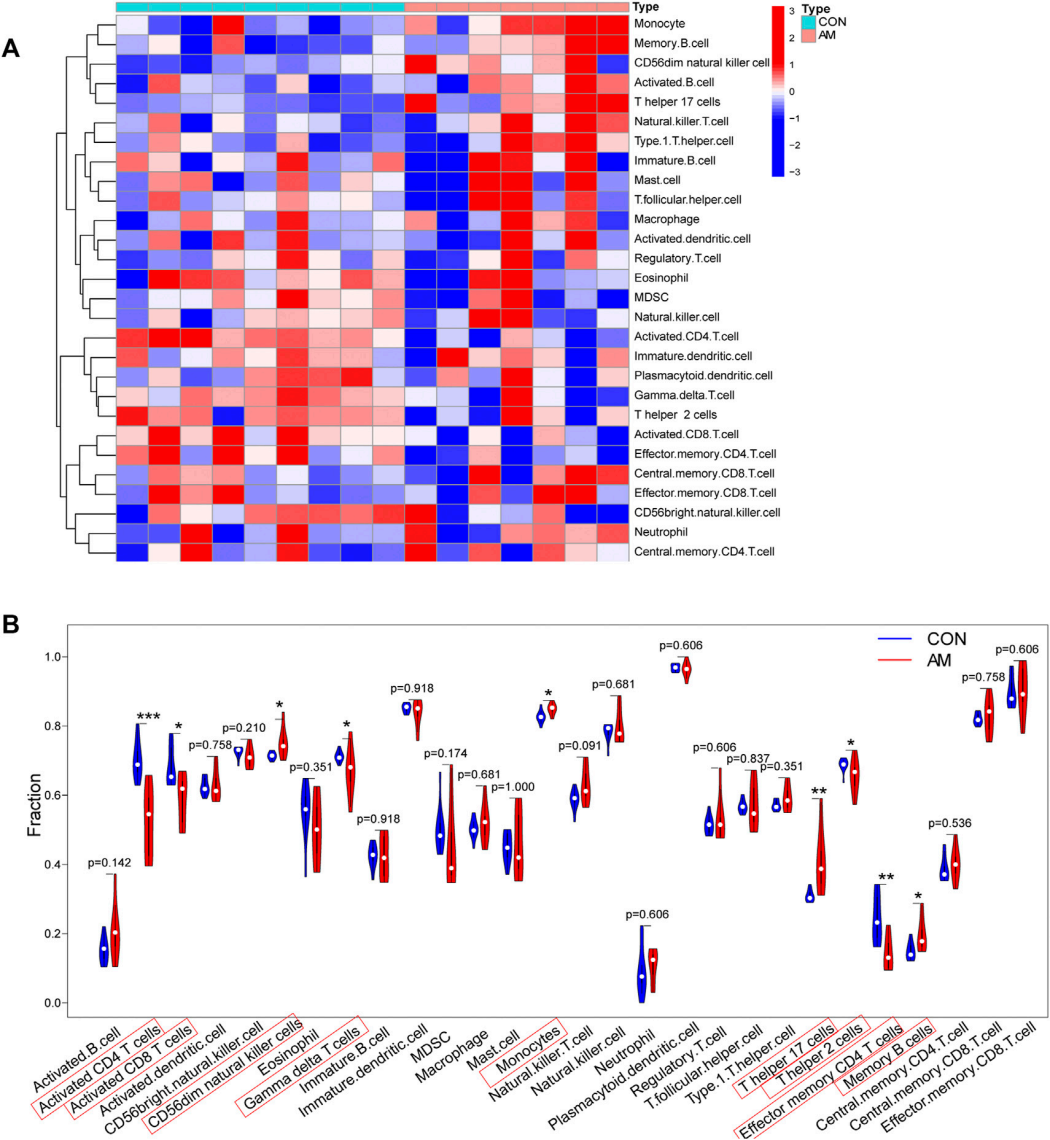
Immune cell infiltration analysis using ssGSEA. **(A)** Heatmap of the distribution of 28 immune cells in the adenomyosis and control group. **(B)** The violin plot of the different distribution of 28 immune cells between the adenomyosis and control groups (*p < 0.05, **p < 0.01, ***p < 0.001, ****p < 0.0001). SsGSEA: Single-sample Gene Set Enrichment Analysis.

To evaluate differences in immune cell infiltration between the endometrium from women with adenomyosis and that from controls, the distribution of 28 immune cells in the expression profile was estimated using ssGSEA. The heatmap of the composition of immune cells in the endometrium samples is shown in Figure 6A. The results indicated that compared with the control group, endometrium from women with adenomyosis had a higher proportion of CD56dim natural killer cells, monocytes, T helper 17 (Th17) cells and memory B-cell but a lower proportion of activated CD4 T-cell, activated CD8 T-cell, gamma-delta T-cell, T helper two cells, and effector memory CD4 T-cell (Figure 6B). These findings suggest a difference in immune infiltration between the endometrium of the adenomyosis and control groups. Thus, CD56dim natural killer cells, monocytes, T helper 17 cells and memory B-cell may have a high correlation with adenomyosis.

### Analysis of correlation between diagnostic markers and infiltration-related immune cells

**FIGURE 7:**
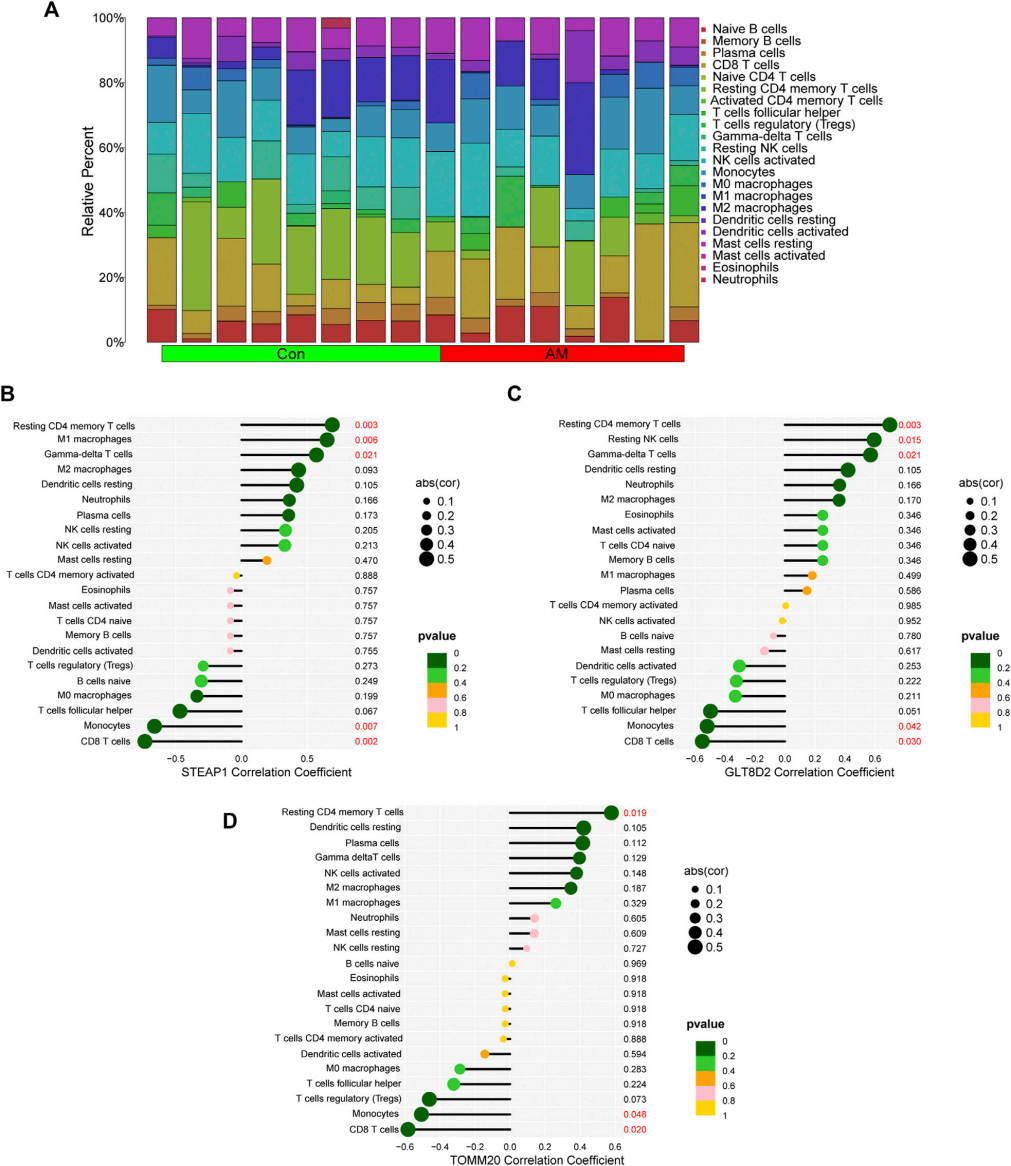
Correlation between diagnostic biomarkers and infiltrating immune cells using CIBERSORT. **(A)** The bar plot of proportion of 22 immune cells in the endometrium of women with adenomyosis and control group analyzed using CIBERSORT. **(B–D)** The correlation between STEAP1, GLT8D2, TOMM20 and infiltrating immune cells. GLT8D2: glycosyltransferase eight domain-containing 2, STEAP1: six-transmembrane epithelial antigen of the prostate-1, TOMM20: translocase of outer mitochondrial membrane 20.

We calculated the proportion of 22 immune cells in all samples using the CIBERSORT algorithm; the results are shown in Figure 7A. We then analyzed the correlation between the infiltration of 22 immune cells and diagnostic markers and found that STEAP1 was positively correlated with the resting CD4 memory T-cell (*p* = 0.003), M1 macrophages (*p* = 0.006), and gamma-delta T-cell (*p* = 0.021) and negatively correlated with monocytes (*p* = 0.007) and CD8 T-cell (*p* = 0.002); GLT8D2 was positively correlated with the resting CD4 memory T-cell (*p* = 0.003), resting NK cells (*p* = 0.015), and gamma-delta T-cell (*p* = 0.021) and negatively correlated with monocytes (*p* = 0.042) and CD8 T-cell (*p* = 0.03); and TOMM20 was positively correlated with resting CD4 memory T-cell (*p* = 0.019) and negatively correlated with monocytes (*p* = 0.048) and CD8 T-cell (*p* = 0.02; Figure 7B). These results indicate that the four biomarkers, namely, STEAP1, GLT8D2, TOMM20, and NME5, may have a high correlation with the dysfunction of immune cell infiltration in adenomyosis.

## Discussion

Adenomyosis is a common gynecological disorder clinically characterized by symptoms such as AUB, severe pelvic pain, and subfertility or infertility, which can affect the health and quality of life of patients (Naftalin et al., 2014; Vercellini et al., 2014). However, the etiology and mechanism of adenomyosis are unclear, and it is important to identify the diagnostic biomarkers of adenomyosis and potential therapeutic targets. Several theories have been proposed including systemic hormonal aberrations, inflammation, and metabolic factors. The most widely accepted theory for the etiology of adenomyosis is the invagination of the basalis endometrium into the myometrium (Bergeron et al., 2006), which indicates the significance of the eutopic endometrium. An abnormal endometrial milieu may contribute to adverse pregnancy outcomes such as miscarriage, recurrent pregnancy loss, and recurrent implantation failure during IVF treatment through hormonal, metabolic, and inflammatory mechanisms (Benaglia et al., 2014; Vercellini et al., 2014; Younes and Tulandi, 2017; Sharma et al., 2019). In addition, women with adenomyosis may be at a high risk of preterm birth and premature rupture of the membrane (Juang et al., 2007). Several studies have indicated aberrant infiltration of immune cells and secretion of inflammatory factors in adenomyosis (Ota et al., 1992; Sotnikova et al., 2002; Yang et al., 2004; Nie et al., 2009). Moreover, the local and systemic immune systems are associated with disease onset and its maintenance (Bourdon et al., 2021).

With the development of next-generation sequencing, transcriptome analysis is becoming an important technique to explore the etiology and mechanism of adenomyosis. Several studies have been performed to identify the gene expression profiles and diagnostic biomarkers of the endometrium from women with adenomyosis using microarray and RNAseq techniques (Herndon et al., 2016; Jiang et al., 2016; Xiang et al., 2019). We formed a new dataset by merging two different GEO datasets and obtained DEGs from the adenomyosis and control samples. Our study identified 318 DEGs, including 33 upregulated and 285 downregulated genes. Subsequent GO and KEGG enrichment analysis showed that DEGs were enriched in nuclear division, mitotic cell cycle phase transition, regulation of mitotic cell cycle, cell cycle, and cellular senescence. These findings indicate dysfunction of the cell cycle, mitosis, proliferation, and cellular senescence in adenomyosis. WGCNA was used to construct gene co-expression networks by clustering genes with similar expression patterns and exploring network modules that are closely associated with clinical traits (Langfelder and Horvath, 2008). This method has been used to identify hub genes in highly connected modules that contribute to diseases (Bakhtiarizadeh et al., 2018). Using WGCNA algorithm and differential expression analysis, we identified 189 genes as adenomyosis-related hub genes by integrating the GSE68870 and GSE78851 datasets. Machine learning algorithms have served as powerful tools to explore the underlying relationships of high-dimensional data and set optimal parameters for gene selection among hub genes with biological significance (Bzdok et al., 2018). In this study, for the first time, we used bioinformatics methods, including WGCNA, LASSO, and SVM-RFE, to identify potential biomarkers of adenomyosis and explore the correlation between infiltrating immune cells and biomarkers. Finally, we screened five biomarkers of adenomyosis. Four biomarkers, namely, STEAP1, GLT8D2, TOMM20, and NME5 were verified using qRT-PCR. ROC analysis demonstrated the accuracy and sensitivity of each biomarker in the diagnosis of adenomyosis. The diagnostic efficacy of the four diagnostic markers combined was also high, as indicated by the ROC curve using logistic regression analysis; however, this needs to be further studied.

The onset and processing of adenomyosis are associated with the immune system. Several observations have highlighted the existence of aberrant immune responses in women with adenomyosis (Rigdon et al., 1987; Sotnikova et al., 2002; Yang et al., 2004; Tremellen and Russell, 2012). One study reported a higher level of Th17 cells in women with adenomyosis than in control women and a relatively low level of regulatory T-cell (Tregs), indicating an imbalance between Th17 cells and Tregs in adenomyosis (Gui et al., 2014). The present study used ssGSEA and CIBERSORT to analyze the immune infiltration of adenomyosis and found that improved infiltration of Th17 cells, CD56dim natural killer cells, monocytes, and memory B-cell may be highly correlated with adenomyosis.

STEAP1 is a member of metalloproteinases family that may participate in iron and copper homeostasis and other cellular processes such as cell proliferation and apoptosis (Xu et al., 2022). The C-terminal domain of STEAP1 is homologous to the *Saccharomyces cerevisiae* ferric reductase. STEAP1 may play a role in attenuating oxidative stress by reducing metal-ion complexes and oxygen by interacting with the NADPH-binding FNO (NADP + oxidoreductase) domain of STEAP2 or STEAP4 (Knutson, 2007; Oosterheert and Gros, 2020). Previous study has shown that STEAP1 is downregulated in endometrial carcinoma and that knockdown of STEAP1 could promote cell proliferation, migration, invasion and epithelial to mesenchymal transition (EMT) (Sun et al., 2019). The downregulation of STEAP1 was also noted in our study in the endometrium of women with adenomyosis, which may lead to abnormal endometrial cell proliferation and EMT induction, both of which play important roles in the etiology of adenomyosis. Furthermore, the dysregulation of STEAP1 may affect the immune infiltration of immune cells and cytokines in different tumors (Zhao et al., 2021; Dorff et al., 2022). In this study, we found that the expression of STEAP1 was positively correlated with the resting CD4 memory T-cell, M1 macrophages and gamma-delta T-cell and negatively correlated with monocytes and CD8 T-cell in adenomyosis. Downregulation of STEAP1 may be related to abnormal immune infiltration in the eutopic endometrium of women with adenomyosis.

TOMM20 is a subunit of the translocase of the outer mitochondrial membrane complex and its function is to recognize and translocate mitochondrial proteins from the cytosol into the mitochondria (Collins, 1976; Yano et al., 2004). The expression of TOMM20 could serve as evidence of active mitochondrial biogenesis and mitochondrial membrane potential. Inactivated mitochondrial biogenesis can lead to mitochondrial dysfunction (Yan et al., 2020). Mitochondria are organelles of the cell respiratory system and provide ATP and ROS. Many studies have indicated that downregulation of TOMM20 could represent mitochondrial dysfunction and is often accompanied with increased oxidative stress (Brown et al., 2019; Nhu et al., 2021; Yamashita et al., 2022). Oxidative stress is associated with various gynecological diseases, including adenomyosis (de Carvalho et al., 2013). In this study, the decreased expression of TOMM20 in adenomyosis indicated the dysregulated mitochondrial function, which can lead to the increased oxidative stress. In addition, TOMM20 was positively correlated with the resting CD4 memory T-cell and negatively correlated with monocytes and CD8 T-cell in adenomyosis. These results indicate a possible connection between the downregulation of TOMM20 and dysfunction of immune cell infiltration in adenomyosis.

GLT8D2 is a member of the glycosyltransferase eight family that contributes to the pathogenesis of non-alcoholic fatty liver disease by regulating the accumulation of triglycerides (Wei et al., 2013). GLT8D2 could contribute to FGFR/PI3K/AKT activation and induce chemoresistance in ovarian cancer (Huang et al., 2021). It may also be involved in the immune system and pathogenesis of human pulmonary artery hypertension (Bai et al., 2021). In our study, the expression of GLT8D2 was downregulated in adenomyosis and positively correlated with the resting CD4 memory T-cell, resting NK cells and gamma-delta T-cell and negatively correlated with monocytes and CD8 T-cell. NME5 is a member of the NME family and contains a conserved domain associated with nucleoside-diphosphate kinase function. NME5 exhibits 3′–5′ exonuclease activity, suggesting its role in DNA proofreading and repair (Puts et al., 2018). Some studies have demonstrated the important role of NME5 in the onset of spermatogenesis (Hwang et al., 2003; Choi et al., 2009; Anderegg et al., 2019). NME5 protected Chinese hamster ovary cells *in vitro* and male haploid germ cells *in vivo* against oxidative stress-induced apoptosis, and its knockdown increased the sensitivity of spermatids in the testes to oxidative stress (Choi et al., 2009). In this study, the expression of NME5 was downregulated in the endometrium of women with adenomyosis, which may lead to oxidative stress. Further experiments are needed to confirm the relationship between biomarkers and the pathogenesis and immune infiltration of the eutopic endometrium in women with adenomyosis.

In this study, we used bioinformatic analysis and machine learning algorithms, including WGCNA, LASSO, and SVM-RFE, to identify four biomarkers of adenomyosis. ssGSEA and CIBERSORT were used to identify differences in immune cell infiltration between the endometrium of women with adenomyosis and that of controls. There are still some limitations in our study. First, the sample size of the datasets collected was small, and the platforms were different. Second, further *in vivo* and *in vitro* experiments are needed to verify the role of diagnostic biomarkers in the pathogenesis and immune infiltration of adenomyosis.

## Conclusion

This study identified STEAP1, TOMM20, GLT8D2, and NME5 as potential biomarkers for adenomyosis. In addition, the presence of Th 17 cells, CD56dim natural killer cells, monocytes, and memory B-cell may be highly correlated with adenomyosis. This provides a new direction for developing new therapeutic targets for adenomyosis.

## Data Availability

The original contributions presented in the study are included in the article/supplementary material, further inquiries can be directed to the corresponding authors.
